# An *in silico* Model of T Cell Infiltration Dynamics Based on an Advanced *in vitro* System to Enhance Preclinical Decision Making in Cancer Immunotherapy

**DOI:** 10.3389/fphar.2022.837261

**Published:** 2022-05-02

**Authors:** Thomas D. Lewin, Blandine Avignon, Alessio Tovaglieri, Lauriane Cabon, Nikolche Gjorevski, Lucy G. Hutchinson

**Affiliations:** Roche Pharma Research and Early Development, Roche Innovation Center, Basel, Switzerland

**Keywords:** cancer immunotherapy, T cell infiltration, *in vitro* cell systems, mathematical modelling, spatio—temporal analysis

## Abstract

Cancer immunotherapy often involves the use of engineered molecules to selectively bind and activate T cells located within tumour tissue. Fundamental to the success of such treatments is the presence or recruitment of T cells localised within the tumour microenvironment. Advanced organ-on-a-chip systems provide an *in vitro* setting in which to investigate how novel molecules influence the spatiotemporal dynamics of T cell infiltration into tissue, both in the context of anti-tumour efficacy and off-tumour toxicity. While highly promising, the complexity of these systems is such that mathematical modelling plays a crucial role in the quantitative evaluation of experimental results and maximising the mechanistic insight derived. We develop a mechanistic, mathematical model of a novel microphysiological *in vitro* platform that recapitulates T cell infiltration into epithelial tissue, which may be normal or transformed. The mathematical model describes the spatiotemporal dynamics of infiltrating T cells in response to chemotactic cytokine signalling. We integrate the model with dynamic imaging data to optimise key model parameters. The mathematical model demonstrates a good fit to the observed experimental data and accurately describes the distribution of infiltrating T cells. This model is designed to complement the *in vitro* system; with the potential to elucidate complex biological mechanisms, including the mode of action of novel therapies and the drivers of safety events, and, ultimately, improve the efficacy-safety profile of T cell-targeted cancer immunotherapies.

## 1 Introduction

In the last decade, cancer immunotherapy (CIT) has emerged as one of the most rapidly advancing and promising fields in the research and development of cancer treatments (Mellman et al., 2011; Couzin-Frankel, 2013; Farkona et al., 2016). Such treatments often involve the use of engineered molecules to selectively bind and activate T cells located within the tumour tissue in order to harness their cytotoxic potential. The presence or recruitment of T cells within the target tissue is crucial to the mode of action of such treatments (Zhang et al., 2019). Thus, a deep understanding of the processes driving T cell trafficking and tissue infiltration and how these are modulated by novel CIT molecules is key to the development of new drugs to reduce the high rate of attrition which results from a lack of efficacy or adverse effects *in vivo* (Havel et al., 2019; Martins et al., 2019). However, observing these dynamics at a high resolution using *in vivo* animal models may be technically challenging. Such models may also fail to be translationally predictive in the clinic as a consequence of genetic differences, with more complex molecules often not cross-reactive with pre-clinical species (Husain and Ellerman, 2018; Olson et al., 2018; Wagar et al., 2018). Aligning with the principle of the “3 Rs” for the refinement, reduction and replacement of animal models (Guhad, 2005), such considerations motivate the development of advanced *in vitro* cell systems towards more controllable, predictive platforms in which to test the pharmacodynamic effects of novel drugs and reduce the emphasis on *in vivo* animal experiments.

We set out to address this gap by building the first *in vitro* system that permits physiologically relevant, basal infiltration of T cells into engineered three-dimensional (3D) intestinal mucosa. We further increased the physiological relevance and predictive capacity of the model by incorporating a resident immune compartment. The multiple components that constitute the model—primary intestinal epithelium, resident immune cells and matched circulating T cells—enable us to introduce controlled modulations that can allow for a simplified representation of variations in patient physiological status within the system, such as chronic inflammation, common asymptomatic infections, microbiome changes, autoimmune susceptibilities or even tissue damage, and immune reprogramming due to cancer or chemotherapy. While T cells are principally responsible for the effects on the epithelium (given the mode of action of TCBs), we cannot exclude the contribution of other immune cell types (B cells, monocytes, NK cells) in the pharmacodynamic effects in both efficacy and safety, even if it is indirect by way of soluble factors, for example. As such, our *in vitro* models incorporate the whole compartment, rather than only T cells (Kerns et al., 2021). Consequently, the experimental data and subsequent analysis presented in this paper consider the infiltration of the PBMC population as a whole.

The control and tractability provided by the platform enables in-depth quantitative analyses of the mechanisms that underlie the observed outcomes. However, the multiple components within the system are interdependent and interact with each other in a complex manner resulting in spatiotemporal dynamics that can be challenging to analyse in a manner that fully utilises the extent of the available data to understand the underlying biology. Mathematical modelling has the potential to enhance the insight gained from such systems and elucidate complex, interrelated biological mechanisms and, ultimately, provide more quantitative predictions. Cellular movement, interactions, and signalling processes have been modelled extensively to mechanistically explore these phenomena using a variety of modelling approaches (DiMilla et al., 1991; Dallon and Othmer, 1997; Matzavinos et al., 2004; Anderson, 2005; Di Costanzo et al., 2015; McLennan et al., 2015). In particular, the chemotactic response has received a lot of attention to understand the directed movement of cell populations and organisms in response to chemical stimuli (Painter et al., 2000; Horstmann, 2003). Among these approaches, variations on the classical system of partial differential equations (PDEs) first formulated by Keller and Segel (1971) have been successfully used to describe the dynamics of a variety of cell populations in different biological contexts (Hillen and Painter, 2009; Painter, 2019). The ability of these systems to mechanistically describe how the distribution of cell populations may evolve spatially and temporally makes them an attractive choice of framework for modelling the rich imaging data which may be collected from advanced *in vitro* cell systems.

There are numerous established techniques which are routinely employed for fitting mathematical models to experimental data. However, these may often be computationally expensive involving large numbers of model simulations; including, but not limited to, Monte Carlo Markov Chain (MCMC) methods, particle swarm optimizers, and differential evolution and genetic algorithms (Storn and Price, 1997; Jin, 2005; Poli et al., 2007; Qin et al., 2009; Gelman et al., 2014). For PDE model systems there are two key considerations which may hinder the use of these approaches, namely, the computational complexity of solving a PDE system numerically and the dimension of the model parameter space to be explored. Surrogate-based optimisation algorithms leverage an approximation of the solution to the full model which may be simulated cheaply in order to perform global optimisation using the aforementioned approaches (Wang and Shan, 2007; Viana et al., 2014). Such methods are routinely used in other fields such as engineering in manufacturing, automotive, and aerospace applications (Wang and Shan, 2007; Laurenceau and Sagaut, 2008; Haftka et al., 2016; Bergh et al., 2020) but their use for complex modelling of biological processes are limited to just a few examples in the literature (Afraites and Bellouquid, 2014; Li et al., 2016; Grenier et al., 2018).

In this paper we present a novel microphysiological system that recapitulates immune cell infiltration into gut epithelial tissue and develop a mathematical model of the *in vitro* system to mechanistically describe the dynamics of infiltrating cells observed experimentally. We combine features from a number of surrogate-based optimisation algorithms and develop a workflow to efficiently explore the model parameter space to fit the model to the spatiotemporal experimental data. We use *in silico* simulations of the mathematical model to analyse the imaging data from the *in vitro* model under a range of experimental conditions to explore how the dynamics of T cell infiltration are altered in the presence of a cytotoxic T cell bispecific antibody (TCB) compared to control, non-toxic conditions.

## 2 Materials and Methods

### 2.1 *In vitro* System

**FIGURE 1:**
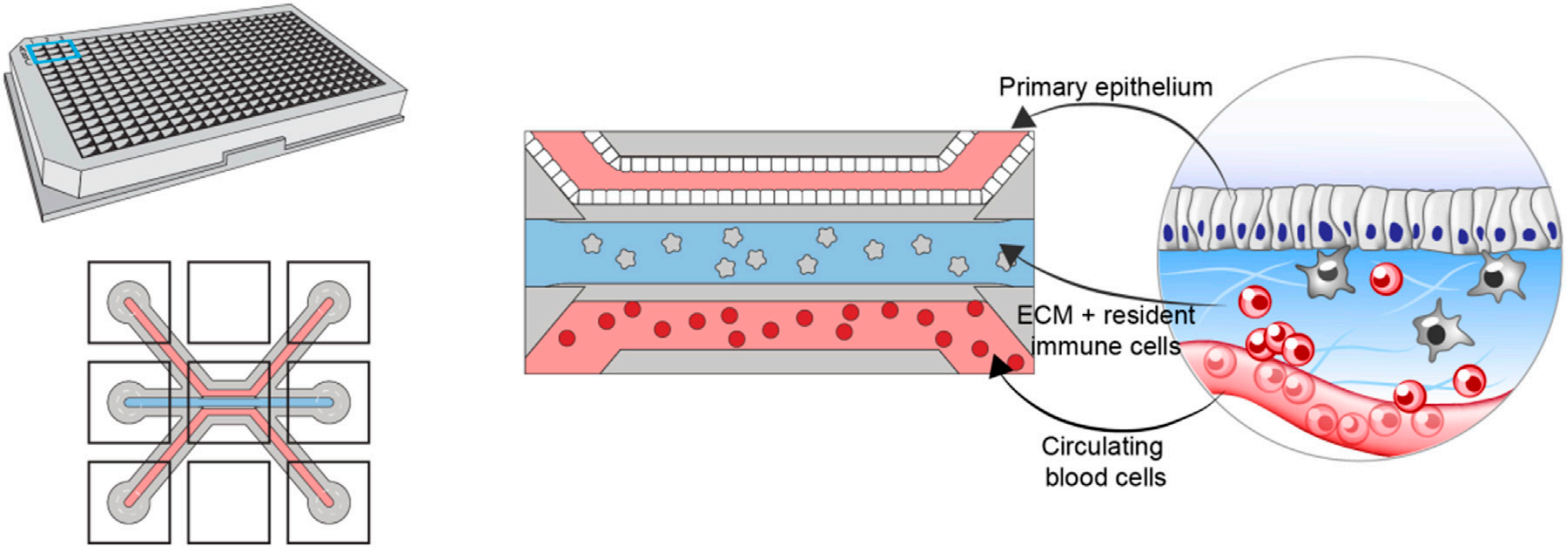
Schematic showing the design and layout of the Mimetas Organoplate chip and the components comprising each of the three channels of each well—primary gut epithelium in the top channel, extracellular matrix and resident immune cells in the middle channel, and peripheral blood mononuclear cells in the bottom channel.

To construct the *in vitro* model, we leverage the multicompartment design of the Organoplate (Mimetas BV, Netherlands)—a microfluidic device containing 40 three-channel chips (Figure 1). The three channels of each chip are delimited *via* a phase guide promoting the formation of distinct microenvironments within each channel while permitting cellular movement between channels [further details of the chip design can be found in (Gjorevski et al., 2020)]. The top (luminal) channel is used to form an intestinal tube using Caco-2 colonocytes. The middle channel is used to create the stromal compartment of the intestine, by incorporating primary monocyte-derived macrophages, embedded in collagen-based extracellular matrix (Gjorevski et al., 2020). The bottom (basal) channel is used to introduce circulating peripheral blood mononuclear cells (PBMCs), including effector T-cells. Importantly, the same pool of PBMCs is used to generate the resident macrophages, thus ensuring matched resident, and peripheral immune compartments.

A tool HLA-restricted T cell bispecific antibody (hereafter toxic TCB), D66-ESK, known to result in broad T-cell mediated killing of HLA-A2-expressing target cells (Augsberger et al., 2021) is used to test whether recreating the cellular and architectural complexity of an immune-responsive intestinal mucosa would allow us to recapitulate the damaging apoptotic effects and investigate the resulting influence on T cell infiltration. A non-targeting, CD3-only binding TCB (hereafter control TCB), DP47, was used as a control expected to yield no toxicity. TCBs, along with PBMCs, were introduced using the basal channel, mimicking systemic delivery in the clinic.

In this paper we present results using resident macrophages that were characterised as an M1 phenotype to provide a pro-inflammatory stimulus and promote T cell migration (Gjorevski et al., 2020). Our dataset comprises 16 different experiments of which 8 were performed in the presence of the control TCB, DP47, and 8 included the cytotoxic TCB, D66-ESK.

The course of each experiment was imaged at 2 h intervals to capture the dynamics of infiltrating PBMCs. Images were acquired using an Opera Phoenix (PerkinElmer) with a 5X objective. The images contain a bright-field and two fluorescent channels (488: Caspase 3/7 green and 555: Cell Tracker red) in a 14 planes z-stack. We analysed the multi-channel images utilizing the Fiji (Schindelin et al., 2012) distribution of ImageJ (Rueden et al., 2017). We opened the images with the Bio formats plugin (Linkert et al., 2010) and reduced the image dimensionality projecting the maximum intensity of the z-planes. We select the area of the image corresponding to the Mimetas Organoplate chip middle and upper compartments utilizing the bright-field channel. To do this we manually defined a region of interest based on the bright-field channel with the ROI-manager tool and cropped the multi-channel images accordingly.

We then quantified the fluorescently labelled immune cells present in the middle and upper chip compartments based on the fluoresce channel. To achieve this we converted the images to a binary mask applying the default auto-threshold method. Additionally, we separated touching nuclei with watershed segmentation. From this segmented image we analysed the particle amount, area, coordinates, and morphology with the Analyze Particles tool. The tabular results were then exported for the *in silico* modelling.

The image analysis algorithm may fail to separate some cells which are closely clumped together. We handle this by incorporating a post-processing step in which cell areas above a threshold size are assumed to be multiple cells occupying the same location, with the number of cells determined by rounding to the nearest multiple of the threshold size. We choose a threshold of 50* μm* ^*2*^ under the assumption that a PBMC is typically less than 10 *μm* in diameter. This is such that regions above 75* μm* ^*2*^ in area ( ∼10μm in diameter) are assumed to comprise more than one cell and rounded up. The spatial distribution of infiltrating PBMCs at each time point is summarised by dividing the region of interest into 20 bins of equal size and counting the number of cells identified in each bin.

### 2.2 Mathematical Model

**FIGURE 2:**
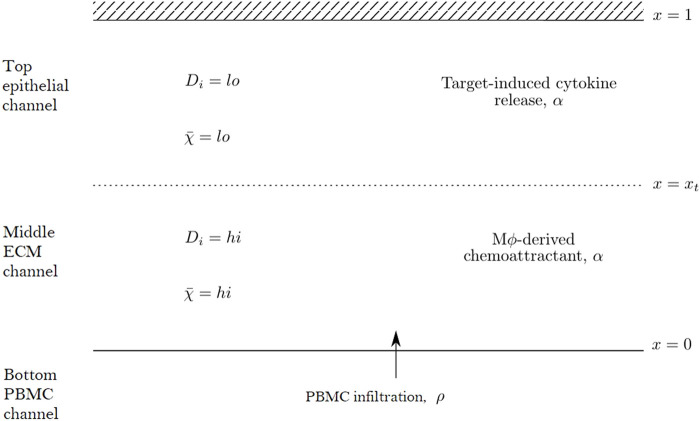
Schematic diagram of the mathematical domain used to model the PBMC infiltration dynamics in the top two channels of the Mimetas Organoplate chip. The interface with the bottom channel is given by *x* = 0, while *x* = *x*_*t*_ denotes the interface between the middle and top channels. The diffusivity parameters, *D*_*i*_, and chemotactic sensitivity, $\tilde{\chi }$ , vary spatially as given by Eq. 4, with cells moving more freely in the middle channel corresponding to higher (hi *vs.* lo) parameter values. Cytokine production may occur in both channels *via* two distinct mechanisms.

We propose a reaction-diffusion-chemotaxis system of partial differential equations (PDEs) to describe the evolution of a continuous PBMC density. The infiltration of PBMCs is assumed to be influenced by the micro-environment in the different channels of the *in vitro* system and responds to cytokine-mediated chemotactic cues. For simplicity, we assume that we may neglect any horizontal movement of PBMCs parallel to the boundaries of each channel and that the infiltration dynamics may be captured considering a single spatial dimension in the forward direction as cells travel across the different channels within the system. We thus describe the dynamics in space, *x*, and time, *t*, of two constituent species within our mathematical model system: the PBMC density, *ρ*(*x*, *t*), and the concentration of a generic chemoattractant, *α*(*x*, *t*). We focus on capturing the dynamics of infiltrating PBMCs and thus consider a domain including the middle matrix channel and top epithelial tissue channel. PBMCs enter the domain at *x* = 0 from the PBMC channel while the boundary *x* = 1 corresponds to the top of the epithelial tissue channel, with the transition between the matrix and tissue channels denoted by *x* = *x*_*t*_ ∈ (0, 1). A schematic representation of this domain is shown in Figure 2.

The reaction-diffusion-chemotaxis system governing the evolution of the PBMC density, *ρ*, and chemoattractant concentration, *α*, may thus be written as:

$$\frac{\partial \rho }{\partial t}=\frac{\partial }{\partial x}\left(D_{\rho }\left(x\right)\frac{\partial \rho }{\partial x}\right)-\frac{\partial }{\partial x}\left(\chi \left(x,\alpha \right)\rho \frac{\partial \alpha }{\partial x}\right)-\xi \rho ,$$ (1)

$$\frac{\partial \alpha }{\partial t}=\frac{\partial }{\partial x}\left(D_{\alpha }\left(x\right)\frac{\partial \alpha }{\partial x}\right)+\underset{\text{Production from macrophages}}{\underset{︸}{\eta_{1}1_{\left\{x\leq x_{t}\right\}}}}+\overset{\text{Target-induced release}}{\overset{︷}{\eta_{2}\rho 1_{\left\{x\geq x_{t}\right\}}}}-\kappa \rho \alpha -\nu \alpha ,$$ (2)

where $1_{A}$ denotes the indicator function on the set *A*.

The interactions between the species in our model describe the dynamics of PBMC infiltration as influenced by a resident immune compartment and the additional effects of drug-target interactions in the epithelial tissue. The production of the chemical species *α* captures the influence of the resident immune compartment in the matrix channel. Here we do not explicitly model the resident macrophages, instead assuming a uniform distribution throughout the matrix and thus uniform rate of production of *α* in 0 < *x* < *x*_*t*_ given by *η*_1_. This mechanism thus represents a pro-inflammatory, macrophage-derived, chemotactic stimulus within our system. If the experimental conditions include the presence of a TCB, for example, then drug-target interactions may occur upon infiltrating PBMCs reaching the tissue channel. This may result in apoptotic epithelial cell death and induce cytokine release, acting as a source of *α* in the top channel. For simplicity we similarly do not model epithelial cell density directly but again assume a uniform distribution such that cytokine release of *α* occurs at a rate, *η*_2_ proportional to the PBMC density, *ρ*, in *x*_*t*_ < *x* < 1. This assumption is of course a simplification and may break down at later times in the case of widespread apoptosis. The cytokines represented by *α* are assumed to degrade with rate *ν* and may be taken up by PBMCs with rate, *κ*. Diffusion throughout the domain is assumed to occur with the spatially varying diffusion coefficient, *D*_*α*_(*x*).

PBMCs are assumed to infiltrate in response to the chemotactic gradient of *α*. Based upon the empirical observations that cytokine expression increases throughout the time course of the experiments while the total number of infiltrating PBMCs saturates, we are motivated to consider a “receptor law” formulation for the chemotactic sensitivity, *χ*(*x*, *α*), given by

$$\chi \left(x,\alpha \right)=\frac{\tilde{\chi }\left(x\right)k}{{\left(k+\alpha \right)}^{2}}.$$ (3)

This well-established and characterised functional form captures the observed behaviour that for large concentrations individual cells may not be able to resolve gradients of the chemoattractant and thus may no longer respond to the chemotactic signal (Painter et al., 2000). In addition to chemotaxis, the cells may also move in an unbiased, diffusive manner. Since we here consider a one-dimensional domain, we also introduce a sink term proportional to the local cell density to account for the small number of cells which are observed to move horizontally and leave the region of interest captured by the image analysis.

Aside from the different mechanisms of cytokine release previously described, we also capture differences between the two channels in the ability of the species to move across each part of the domain. More specifically, it is assumed that cells may move and cytokines may diffuse more freely through the matrix channel than they may penetrate the epithelial tissue. These differences manifest themselves in the form of piece-wise constant diffusion and chemotaxis coefficients, *D*_*ρ*_(*x*), *D*_*α*_(*x*) and $\tilde{\chi }\left(x\right)$ , which are defined by

$$D_{\rho }\left(x\right)=\left\{\begin{array}{cc} {\bar{D}}_{\rho }\; & 0<x<x_{t}\\ {\bar{D}}_{\rho }/\mu_{\rho }\; & x_{t}<x<1, \end{array}\right.\;D_{\alpha }\left(x\right)=\left\{\begin{array}{cc} {\bar{D}}_{\alpha }\; & 0<x<x_{t}\\ {\bar{D}}_{\alpha }/\mu_{\alpha }\; & x_{t}<x<1, \end{array}\right.\;\tilde{\chi }\left(x\right)=\left\{\begin{array}{cc} \bar{\chi }\; & 0<x<x_{t}\\ \bar{\chi }/\mu_{\rho }\; & x_{t}<x<1, \end{array}\right.$$ (4)

for *μ*_*ρ*_, *μ*_*α*_ > 1, where ${\bar{D}}_{\rho }$ , ${\bar{D}}_{\alpha }$ and $\bar{\chi }$ are scalar parameters for the motility coefficients. Thus the diffusion and chemotaxis coefficients are higher (c.f. “hi” in Figure 2) in the middle channel than in the top channel (c.f. “lo” in Figure 2).

It remains to specify appropriate initial and boundary conditions to close the system given by Eqs. 1, 2. For the boundary at the top of the tissue channel at *x* = 1 we prescribe no flux conditions for *ρ* and *α* given by:

$${\left.D_{\rho }\frac{\partial \rho }{\partial x}-\chi \frac{\partial \alpha }{\partial x}\rho \right|}_{x=1}=0,\;{\left.\frac{\partial \alpha }{\partial x}\right|}_{x=1}=0.$$ (5)

The boundary at *x* = 0, however, does not represent a solid boundary of the *in vitro* system, but rather the interface between the bottom PBMC channel and the middle compartment representing the ECM. As a consequence of the experimental observation that very few PBMCs infiltrate in the absence of macrophages in the system, we assume that the initial infiltration of PBMCs into the matrix channel is driven by chemotaxis. The total flux of PBMCs into the domain at *x* = 0 is thus proportional to the chemotactic signal from *α* which may be written as:

$$-D_{\rho }\frac{\partial \rho }{\partial x}+\chi \frac{\partial \alpha }{\partial x}\rho =\chi \frac{\partial \alpha }{\partial x}\tilde{\rho },$$ (6)

where $\tilde{\rho }$ represents the assumed constant source of PBMCs in the bottom channel. The cytokine species *α* is able to diffuse across the interface at *x* = 0 and so we prescribe a diffusive flux out of the domain proportional to the concentration such that

$$\frac{\partial \alpha }{\partial x}=\zeta \alpha .$$ (7)

The *in vitro* system is initialised with the pool of PBMCs contained within the bottom channel. The resident immune compartment is present at the start of the experiment and provides the chemotactic stimulus for PBMC infiltration. We therefore assume a non-zero, steady state initial condition for the concentration of *α* due to the production by the macrophages in the matrix channel. Initial conditions for the PBMC density, *ρ*, and the cytokine concentration, *α*, throughout the domain are thus given by:

$$\rho \left(x,0\right)=0,\;\alpha \left(x,0\right)=\bar{\alpha }\left(x\right),$$ (8)

where $\bar{\alpha }\left(x\right)$ satisfies

$$\frac{\partial }{\partial x}\left(D_{\alpha }\left(x\right)\frac{\partial \bar{\alpha }}{\partial x}\right)+\eta_{1}1_{\left\{x\leq x_{t}\right\}}-\nu \bar{\alpha }=0.$$ (9)

Eqs. 1–9 completely describe our spatiotemporal mathematical model of PBMC infiltration in the *in vitro* system. We note that this model is similar to that proposed by Alt and Lauffenburger (Lauffenburger and Kennedy, 1983; Alt and Lauffenburger, 1987) for modelling T cell infiltration *in vivo*. We simulate the solution of our model using a finite volume numerical scheme, of which more details may be found in the Supplementary Material.

### 2.3 Parameter Optimisation Framework

In order to integrate the mathematical model developed in Section 2.2 with the *in vitro* experimental data of PBMC infiltration, we require a framework to efficiently explore the model parameter space. In this section we present a summary of the key ideas of the optimisation methodology used in this paper, which is summarised by the pseudo-code in Algorithm 1. Please see the Supplementary Material and the references therein for a more detailed explanation.

Kriging, also known as Gaussian process modelling, is a method of statistically interpolating data to build a response surface (Sacks et al., 1989). First introduced by Jones et al. (1998) based on ideas developed by Sacks et al. (1989), the key idea of surrogate-based optimisation is to leverage the Kriging surrogate for computationally-intensive global optimisation to minimise the number of calls to numerically simulate the true function. In brief, a typical Kriging-based optimization algorithm proceeds by first building an initial Kriging model based on a random sample of the parameter space [e.g., Latin hypercube sample (LHS)]. Subsequent iterations involve the use of a differential evolution (DE) global optimisation algorithm (Storn and Price, 1997) on the Kriging model to identify the best next point to sample based on a metric of expected improvement (EI) over the current minimum. The true model is then solved at the identified point and the Kriging model correspondingly updated. The algorithm iterates until either a convergence tolerance is met or a pre-determined computational budget is exceeded.

In our framework, we incorporate additional features from a number of existing algorithms to improve the speed and convergence of the algorithm to the global optimum. These features include parallelisation (Zhan et al., 2017), additional sampling criteria (Sacher et al., 2018; Xing et al., 2020), and domain size reduction (Xing et al., 2020).

Algorithm 1 Kriging-based optimisation workflow.
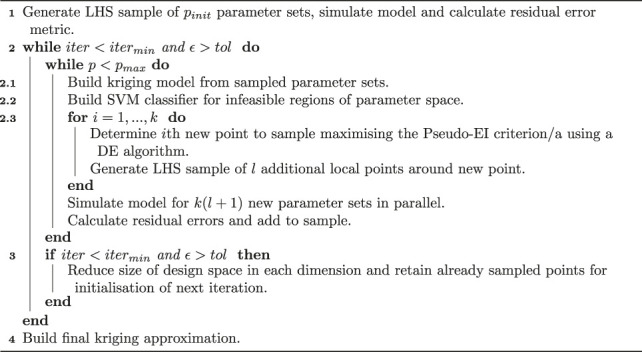


The PBMC distribution at each time point is characterised from the *in vitro* data by splitting the domain into equally sized bins and counting the number of cells identified in each. Correspondingly, we also numerically integrate the solution for PBMC density across each bin. We calculate the sum of squares distance between each bin count and the solution summed over all time points to give the distance metric to be minimised as the objective function for the optimisation algorithm.

## 3 Results

### 3.1 Recapitulating T Cell Infiltration *in vitro*

**FIGURE 3:**
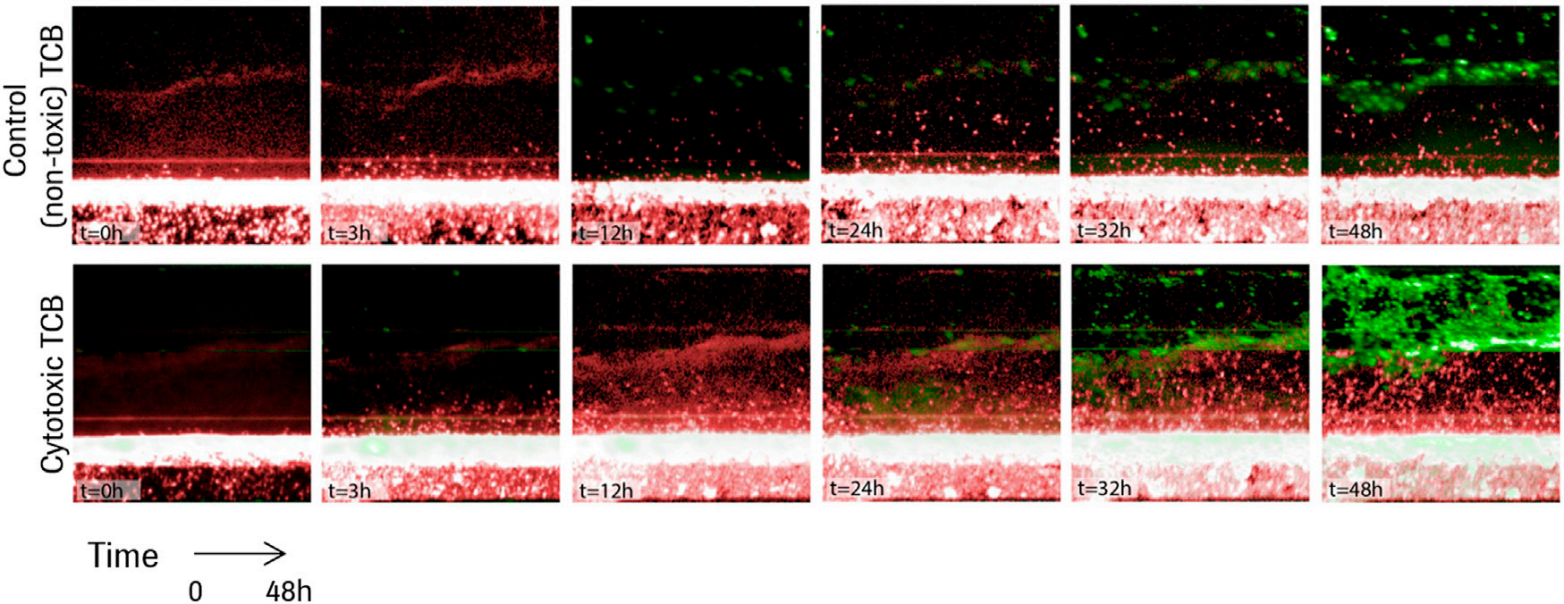
Time lapse images for the first 48 h of two representative experiments in the presence of a control TCB, DP47 (top), and in the presence of a cytotoxic TCB, D66-ESK (bottom). Infiltrating PBMCs are tracked in red while apoptotic epithelial cells are imaged in green.

A series of time lapse images for two representative experiments are shown in Figure 3, one in the presence of the non-toxic, control TCB, DP47, and one in the presence of the cytotoxic TCB, D66-ESK. A video of the time lapse imaging for an experiment in cytotoxic TCB conditions can be found in the online Supplementary Material. From a visual comparison, it is evident that the *in vitro* system recapitulates differences in both PBMC infiltration and epithelial cell apoptosis between the two conditions. Soon after TCB treatment, as observed in the snapshot at 3 h in Figure 3, lymphocytes began infiltrating the ECM compartment, likely also guided by cytokines produced by the resident macrophages. Within 48 h, however, toxic TCB treatment resulted in substantially higher PBMC infiltration compared with the control TCB. Moreover, in the toxic TCB-treated *in vitro* model, PBMC infiltration culminated with massive epithelial cell killing *via* apoptosis, which is consistent with mechanisms of T cell cytotoxicity.

**FIGURE 4:**
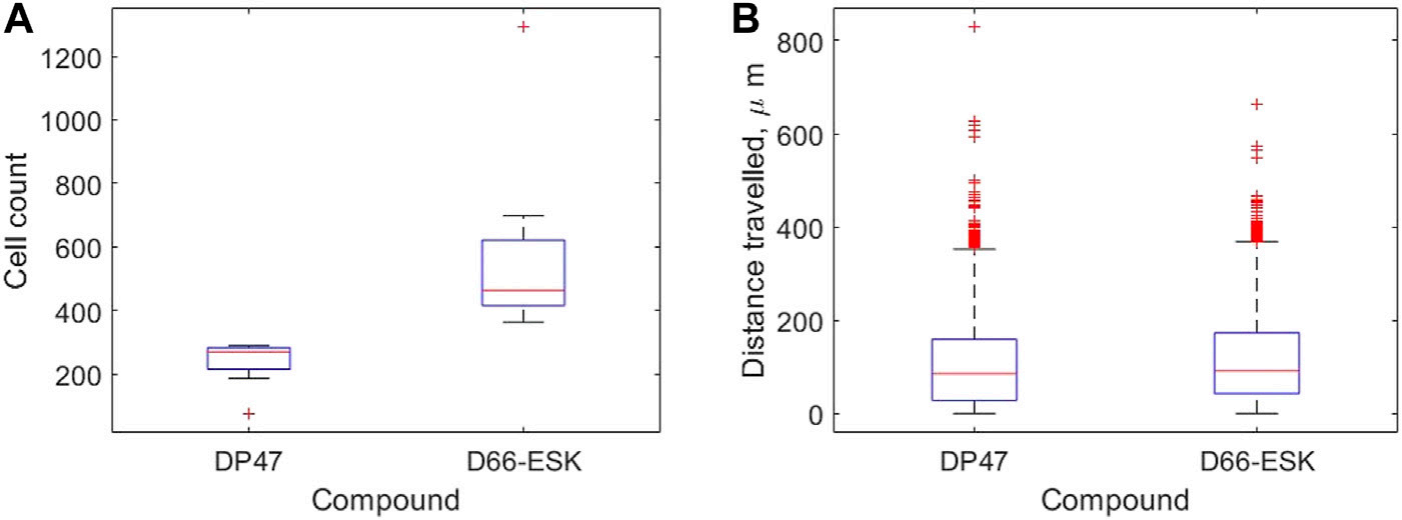
Summaries of PBMC infiltration data at 48 h grouped by compound. **(A)** The total number of infiltrating cells. **(B)** The distribution of distances travelled per infiltrating cell away from the interface with the bottom channel in *μ*m.

We use our image analysis pipeline to identify infiltrating PBMCs and quantify precisely these observations. We visualise how the extent of PBMC infiltration at 48 h differs between the control TCB and cytotoxic TCB conditions in Figure 4. In Figure 4A we confirm that significantly more PBMCs have migrated into the top two channels after 48 h in conditions in the presence of the cytotoxic TCB. Across the 16 experiments in our dataset, a mean of 582.13 cells are identified in the D66-ESK conditions compared to 237.63 cells in the DP47 conditions. However, if we visualise how these populations of infiltrating PBMCs are distributed throughout the system we observe very little difference between the two conditions (Figure 4B). The majority of infiltrating cells are spread throughout the middle ECM channel with a mean distance travelled of 109.81 and 119.20 *μ*m for the DP47 and D66-ESK conditions, respectively. The leading cells that travel the furthest and reach the interface with the top epithelial channel at 350 *μ*m appear to cluster close to the interface and do not significantly penetrate through this epithelial barrier.

### 3.2 Mathematical Model Fitting to Data

**FIGURE 5:**
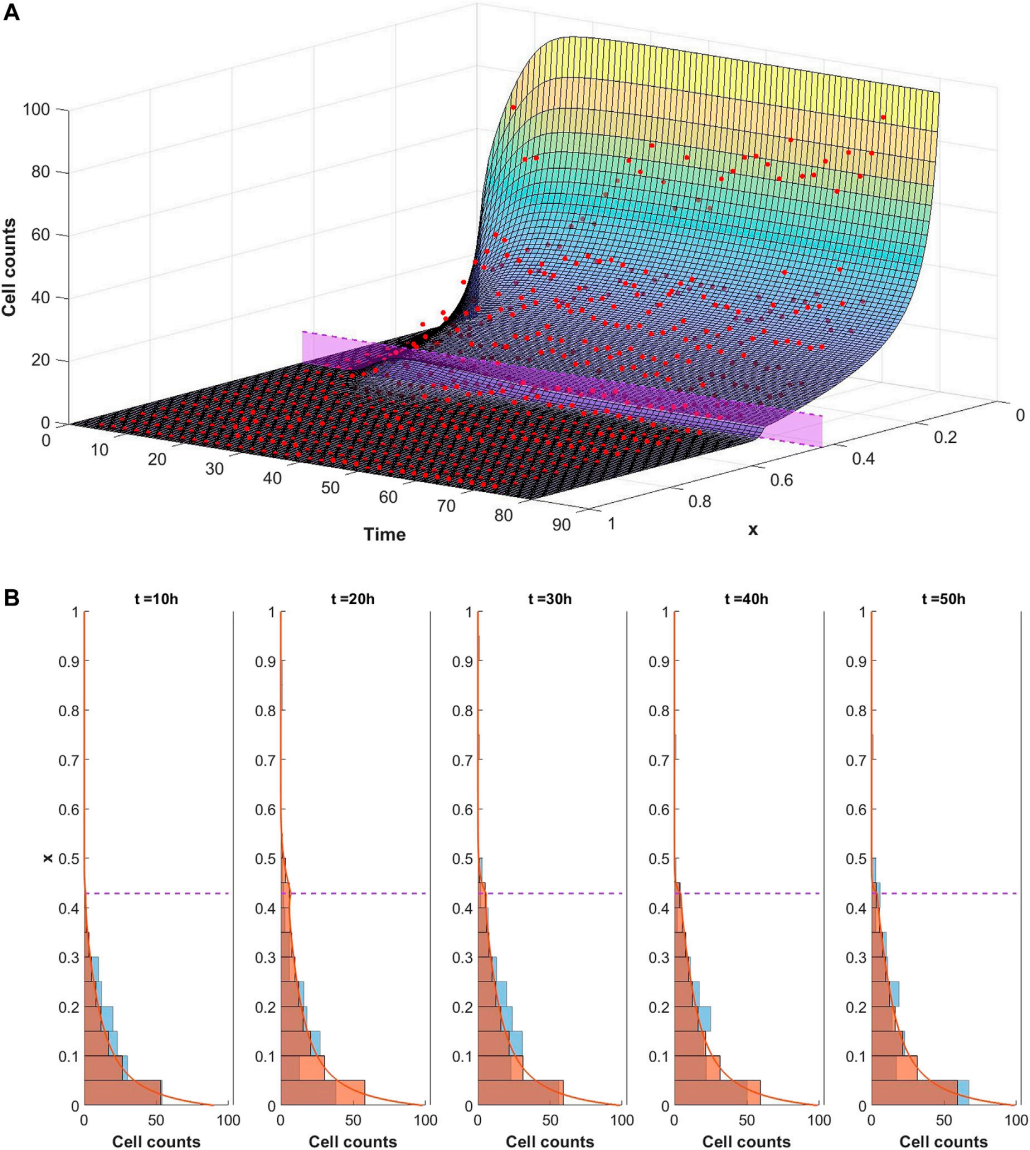
**(A)** Visualisation of the mathematical model exhibiting a good fit to the experimental data for conditions with 10 *μ*g/ml of DP47. The cell counts for each bin at each time point are shown by the red dots. The model is simulated up to 80 h using the optimal parameters found using Algorithm 1 (given in Supplementary Table S2 in the supplementary material). The solution surface for the PBMC density, *ρ*, is scaled to account for the bin width and overlaid with the data points. The purple region denotes the position of the interface between the middle and top channels of the *in vitro* system. **(B)** Comparison of simulated cell count distributions (red bars) in 10 h intervals with experimental data (blue bars) for the same data and simulation shown in Figure 5. The scaled simulated PBMC density *ρ* is overlaid for reference (red line).

The ability of the mathematical model given by Eqs. 1–9 to describe the observed dynamics is assessed by fitting the model to the experimental data. We consider each well of the plate to be a different experiment and separately optimise the model fit to each dataset in turn. In each case the optimisation framework described in Section 2.3 is used to identify the parameter set which best describes the data. An example of a good fit to the data is shown in Figure 5 whereby the model well describes the dynamics of infiltrating PBMCs *in vitro*. In this particular case the experiment was performed in the presence of 10 *μ*g/ml of the control TCB, DP47. The surface in Figure 5A represents the model solution for the PBMC density, *ρ*, as it evolves in space and time. The red points summarising the cell counts of infiltrating PBMCs identified in the imaging data lie close to the solution surface and are evenly distributed around it. From the model solution we can see that the infiltration dynamics comprise an initial infiltration phase, lasting ∼24 h, during which PBMCs infiltrate the system, migrate through the middle ECM channel and begin to accumulate at the interface with the epithelial tissue. The first cells reach the interface with the epithelial cell channel and start to accumulate around 12–15 h into the experiment. Subsequently, a steady state distribution profile is reached with few cells penetrating the epithelial channel beyond the cellular interactions at the interface. A more granular time lapse showing the comparison between the simulated cell distributions and the data is shown in Figure 5B.

**FIGURE 6:**
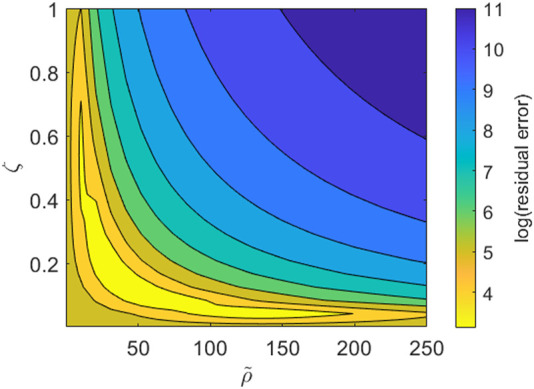
Contour plot for the parameter subspace of the pool size of PBMCs, $\tilde{\rho }\in \left[0,250\right]$ , and the cytokine outflux rate, *ζ* ∈ [0, 1]. The contours represent the quality of fit to the data shown in Figure 5 as given by the sum of squares residual error and is shown on a log scale. All other model parameters are fixed to those used for the simulation in Figure 5.

We note, however, that while our imaging dataset provides good resolution on the PBMC infiltration dynamics throughout the time course of the experiment, we lack information on the cytokine expression in each channel and how it changes over time. Consequently some model parameters are not identifiable with respect to the current dataset. This is most obviously seen when we consider the boundary conditions given by Eqs. 6, 7. Using Eq. 7, the right hand side of Eq. 6 becomes $\chi \left(0,\alpha \right)\zeta \tilde{\rho }\alpha$ . Consequently, in the absence of cytokine data, we would anticipate that the parameters *ζ* and $\tilde{\rho }$ are not identifiable. We may verify this by fixing all other model parameters to those used in Figure 5 and exploring the $\left(\tilde{\rho },\zeta \right)$ -subspace. In Figure 6 we visualise the contours of this subspace with respect to model fit to the data. As might be expected from the equations, we observe a reciprocal relationship between $\tilde{\rho }$ and *ζ* which gives rise to parameter combinations with equally good fits.

### 3.3 The Influence of Cytotoxic TCBs

**FIGURE 7:**
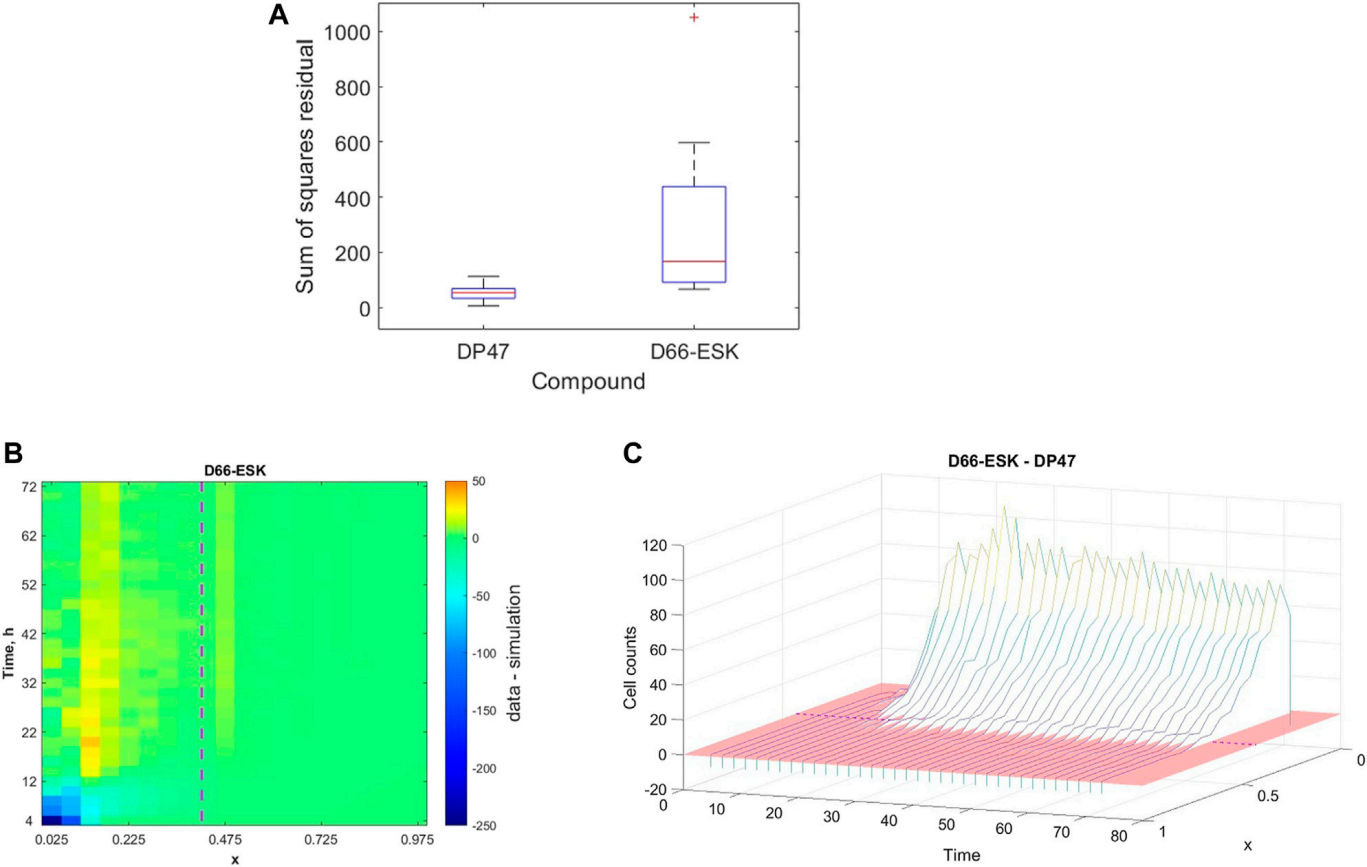
**(A)** Boxplots showing the distribution of the best model fits to the data across all experimental conditions separated by compound between the control TCB, DP47, and the cytotoxic TCB, D66-ESK. **(B)** Heatmap of the discrepancy between the model simulations and the data for each data point aggregated across all conditions in the presence of D66-ESK. The purple dashed line marks the position of the interface between the middle and top channels. **(C)** Waterfall plot showing the difference in distribution of infiltrating PBMCs at each time point between the D66-ESK conditions and the DP47 conditions. The red region shows the plane where the difference in cell count is zero. The location of the interface between the middle and top channels of the plate is marked by the purple dashed line.

While we may not make any concrete statements based on the identified parameter values arising from our parameter fitting as a result of the unidentifiability of a number of model parameters, we may still more broadly interpret the ability of the mathematical model and the proposed mechanisms to describe the *in vitro* infiltration dynamics in the data based on the quality of fit. In Figure 5, we presented an example of data from a single experiment to which the model provides a good fit. However, as might be expected, there is variability in both the experimental data and, correspondingly, the quality of the model fit to the data. Our dataset includes experiments performed under a variety of experimental conditions, in particular in the presence of either a control, non-toxic TCB, DP47, or a cytotoxic TCB, D66-ESK. In Figure 7A we visualise the results of the parameter optimisation grouped by compound. When grouped by compound we observe that the mathematical model consistently describes the conditions in the presence of the control TCB, DP47, better than those treated with the cytotoxic TCB, D66-ESK, in absolute terms.

To further investigate the discrepancies between the mathematical model and the data in the D66-ESK treated conditions we consider each residual to the data in space, *x*, and time, *t*. We sum the values for each residual across all experiments performed in the presence of D66-ESK and visualise the results as a heatmap in (*x*, *t*)-space in Figure 7B. The dark blue area of the heatmap near (*x*, *t*)=(0,0) corresponds to the initial infiltration phase with large negative residuals corresponding to a significant overestimation of the early dynamics by the model simulations. By contrast, the predominantly green colours at later times represent a relatively good fit of the model to the data. As discussed in Section 3.1, the presence of D66-ESK results in an increase in the number of infiltrating PBMCs compared to the DP47 conditions. In order to optimise the fit to the data, the model simulations closely match the distribution profile at later times when more cells are present in the system at the expense of capturing the initial infiltration phase. The inability of the model to capture both phases with a single parameter set suggests that the increased infiltration observed with the cytotoxic TCB does not arise as simply an amplification of the mechanisms present in the control TCB conditions.

The distinction between the dynamics observed in the presence of the cytotoxic TCB versus the control TCB is further highlighted when we plot the difference between the aggregated D66-ESK data points and the aggregated DP47 data points (Figure 7C). It is evident that there is very little difference in the early dynamics between the two conditions with a notable increase in infiltration in the cytotoxic TCB conditions occurring after approximately 12 h. We note that this discrepancy appears to coincide with the progress of the first infiltrating cells. This suggests that the second, increased infiltration phase in the D66-ESK dynamics may result from the effects of target engagement by the first infiltrating PBMCs mediated by a chemotactic signal that is distinct from that provided by the resident macrophages in the ECM channel. Consequently, PBMCs are able to first moderately infiltrate in response to a pro-inflammatory stimulus, and subsequently increase infiltration upon target engagement.

## 4 Discussion

In this paper, we have presented a novel microphysiological system which recapitulates the dynamics of infiltrating T cells into tissues. The *in vitro* model includes the effects of a resident immune compartment and exhibits observable differences in the dynamics in the presence of different immune modulatory compounds. The multiple components that constitute the *in vitro* model—primary intestinal epithelium, resident immune cells and matched circulating T-cells—enable us to introduce controlled modulations that can allow for a simplified representation of variations in patient physiological status within the system, such as chronic inflammation, common asymptomatic infections, microbiome changes, autoimmune susceptibilities or even tissue damage, and immune reprogramming due to cancer or chemotherapy. Thus, we created a complex immune-competent model of the intestine, and the first to incorporate T-cell infiltration as a crucial step of the cancer immunity cycle. We also demonstrated the model’s competence to recapitulate TCB-mediated T-cell activation and epithelial cell killing. As such, our system provides a highly promising setting in which to test new drugs and provide predictions for safety and efficacy *in vivo*.

Live imaging of the *in vitro* system allows for the observation of such dynamics at a high temporal resolution. Image analysis techniques can provide a rich dataset for quantitative analysis of the complex biological processes involved. Simple analysis of the imaging data confirmed greater infiltration of PBMCs was oberved in the presence of a cytotoxic TCB at 48 h when compared to control, non-toxic conditions. No significant difference, however, was observed in the distribution of distances travelled by infiltrating PBMCs between the two conditions. In both scenarios, infiltrates were spread throughout the middle matrix channel with cells observed to accumulate upon reaching the interface with the epithelial cells. When in the presence of the cytotoxic TCB this target-engagement with the epithelial cells was observed to trigger apoptosis.

However, reduction of the complete, spatiotemporal dataset to summary statistics in this manner, does not utilise the data to its full extent or maximise the insight which may be gained into the spatiotemporal dynamics. To that end, we developed a mathematical model to describe the infiltration of PBMCs in response to chemotactic signals pertaining to the particular geometry and components of our *in vitro* system. We implemented a surrogate-based optimisation algorithm in order to fit the model to the experimental data. We observed that the model may provide a good description of the PBMC infiltration dynamics and can characterise an initial infiltration phase that subsequently settles to a steady state distribution of PBMCs throughout the system whereby infiltrating cells are spread throughout the middle, matrix channel but do not significantly penetrate the top, epithelial tissue channel.

Inspection of the model equations and subsequent analysis of a subspace of the full parameter space revealed that the model is not practically identifiable with respect to the current imaging data. This limits the extent to which we may make quantitative conclusions about specific parameters and the magnitude of influence of different processes. We thus have identified that additional data is necessary to further inform the mathematical model in order to ultimately make more quantitative predictions. In particular, the ability to robustly estimate the model parameters may allow for further, quantitative understanding for the TCB dose dependence on the infiltration dynamics, for example. Although not available for the experimental data presented here, it is feasible to measure cytokine readouts at discrete timepoints within the top and bottom channels of each well. As such, the use of the current model to inform the design of future experiments is an important avenue for future work. The model may be used to inform the types of data which should be measured as well as identifying the most informative time points at which to sample. This is of particular relevance for cytokine readouts which may not be continuously monitored as for the imaging data and are therefore more costly to sample.

By analysing the quality of fit of the mathematical model to the data we identified differences between the conditions in the presence of a cytotoxic TCB, D66-ESK, versus a control, non-toxic TCB, DP47. The discrepancies between the model and data with the cytotoxic TCB do not arise simply as a result of uniformly increased numbers of infiltrating PBMCs. Under the hypothesised mechanisms described by the model, in order to achieve the large numbers of infiltrating PBMCs at steady state at later times, the initial infiltration phase must also necessarily be accelerated. This is evident in the heatmap of the residuals in the cytotoxic TCB conditions whereby the model fit is compromised with a significant overestimation of the initial dynamics in order to accomodate the steady state profile. Further analysis of the differences between the control and cytotoxic TCB conditions showed that in both cases the initial phase is almost identical, with acceleration of the dynamics in the cytotoxic TCB case occurring after ∼10–12 h coincident with the first PBMCs reaching the interface with the epithelial cell channel. The relatively poor fit of the model to these dynamics suggests that the cytokine signalling as a result of this target engagement must act as a distinct chemotactic cue for the infiltrating PBMCs, rather than as an amplification of the existing, initial chemotactic trigger provided by the resident macrophages. Extending the model to account for these mechanisms will further increase the complexity of the system. An exploration of these model dynamics for the cytotoxic TCB conditions is an important area of future work in combination with further experiments to supplement data available for model calibration as discussed above.

In this paper we focus on PBMC infiltration dynamics towards epithelial tissue and thus, for simplicity, we model the cellular distribution in a single spatial dimension perpendicular to the direction of each channel of the *in vitro* system. The model equations naturally generalise to higher dimensions, and simulations of such are an avenue for future work in order to validate the work presented in this paper and subsequent extrapolation to geometries beyond that imposed by the *in vitro* system considered here. By contrast, typical pharmacokinetic/pharmacodynamic (PKPD) models used in drug development context are often formulated as systems of ODEs. It is likely feasible to reduce the mathematical model in this paper to an ODE description of the total number of infiltrating PBMCs in the manner presented by Alt and Lauffenburger (1987). Such an analysis would facilitate integration of these dynamics with commonly-used PKPD modelling frameworks.

The *in vitro* system, experimental data, mathematical model and analysis presented in this paper may be used provide insight and understanding of the spatiotemporal dynamics of PBMC infiltration and how they are influenced by novel immune-modulatory compounds. This is of fundamental interest for understanding the interrelated signalling mechanisms involved. However, perhaps of more importance for drug development, is the potential for these systems to predict the consequent apoptosis induced by target-engagement with these compounds in both a safety and an efficacy context. Thus, an important future extension of the current modelling will be to incorporate a description of the epithelial cell population to investigate the influence of the spatiotemporal infiltration dynamics on target-mediated cell death with the aim of quantitative *in vivo* predictions for the safety and efficacy of novel cancer immunotherapy drugs.

In conclusion, mathematical approaches to modelling *in vitro* systems, such as the one presented in this paper, can aid in the design and analysis of complex experiments representing *in vivo* biology, provide insights into interrelated biological mechanisms and, ultimately, provide more quantitative predictions to develop safe, efficacious drugs.

## Data Availability

The raw data supporting the conclusion of this article will be made available by the authors, without undue reservation.

## Supplementary Material

Supplementary Video S1 Time lapse of in vitro PBMC infiltration in the presence of a cytotoxic TCB, D66-ESK. Infiltrating PBMCs are tracked in red while apoptotic epithelial cells are imaged in green.
