# Supplementary material for: An in silico Model of T Cell Infiltration Dynamics Based on an Advanced in vitro System to Enhance Preclinical Decision Making in Cancer Immunotherapy
Source: Front Pharmacol. 2022 May 2;13:837261. doi: 10.3389/fphar.2022.837261 (PMC9108393; doi:10.3389/fphar.2022.837261)
Supplement: Supplementary file 2 [file DataSheet1.PDF]

Supplementary material - An *in silico* model of T cell infiltration dynamics based on an advanced *in vitro* system to enhance preclinical decision making in cancer immunotherapy

Thomas D. Lewin[1], Blandine Avignon[1], Alessio Tovaglieri[1], Lauriane Cabon[1], Nikolche Gjorevski[1], and Lucy G. Hutchinson[1]

[1]*Roche Pharma Research & Early Development, Roche Innovation Center, Basel, Switzerland*

1 Numerical scheme

The mathematical model for T cell infiltration given by Equations (1)-(9) in the main text is solved numerically using a finite volume method. We discretise the spatial domain into N equally sized volumes with a mesh size Δx, and use a timestep Δt for the temporal discretisation. We denote by ρ_i^n and α_i^n the average values of the variables ρ and α, respectively, in cell i at the nth timestep.

We solve Equation (3) for the T cell density ρ in cells $i = 2, ..., N-1$ using an implicit scheme for the diffusion term and upwinding for the advection term:

$$\begin{aligned}\frac{\Delta x}{\Delta t}\left(\rho_i^{n+1}-\rho_i^n\right) - D_{i+\frac{1}{2}}\frac{\left(\rho_{i+1}^{n+1}-\rho_i^{n+1}\right)}{\Delta x} + D_{i-\frac{1}{2}}\frac{\left(\rho_i^{n+1}-\rho_{i-1}^{n+1}\right)}{\Delta x}\\ + \frac{1}{2}\chi_{i+\frac{1}{2}}6n\frac{\left(\alpha_{i+1}^n-\alpha_i^n\right)}{\Delta x}\left(\rho_{i+1}^n-\rho_i^n\right) - \frac{1}{2}\chi_{i+\frac{1}{2}}^n\frac{\left|\alpha_{i+1}^n-\alpha_i^n\right|}{\Delta x}\left(\rho_{i+1}^n-\rho_i^n\right)\\ - \frac{1}{2}\chi_{i-\frac{1}{2}}^n\frac{\left(\alpha_i^n-\alpha_{i-1}^n\right)}{\Delta x}\left(\rho_i^n-\rho_{i-1}^n\right) + \frac{1}{2}\chi_{i-\frac{1}{2}}^n\frac{\left|\alpha_i^n-\alpha_{i-1}^n\right|}{\Delta x}\left(\rho_i^n-\rho_{i-1}^n\right) = \Delta x\xi\rho_i^n, \qquad \text{(S1)}\end{aligned}$$

where

$$D_{i\pm\frac{1}{2}} = \frac{2D_iD_{i\pm1}}{D_i+D_{i\pm1}} \qquad \text{and} \qquad \chi_{i\pm\frac{1}{2}}^n = \frac{2\chi(x_i,\alpha_i^n)\chi(x_{i\pm1},\alpha_{i\pm1}^n)}{\chi(x_i,\alpha_i^n)+\chi(x_{i\pm1},\alpha_{i\pm1}^n)}. \qquad \text{(S2)}$$

The boundary condition at $x = 0$ (Equation (6)) is implemented in cell 1 as:

$$\begin{aligned}\frac{\Delta x}{\Delta t}\left(\rho_1^{n+1}-\rho_1^n\right) - D_{\frac{3}{2}}\frac{\left(\rho_2^{n+1}-\rho_1^{n+1}\right)}{\Delta x} + \frac{1}{2}\chi_{\frac{3}{2}}^n\frac{\left(\alpha_2^n-\alpha_1^n\right)}{\Delta x}\left(\rho_2^n-\rho_1^n\right) - \frac{1}{2}\chi_{\frac{3}{2}}^n\frac{\left|\alpha_2^n-\alpha_1^n\right|}{\Delta x}\left(\rho_2^n-\rho_1^n\right)\\ = \Delta x\xi\rho_i^n + \tilde{\rho}\chi_1^n\zeta\alpha_1^n. \qquad \text{(S3)}\end{aligned}$$

Correspondingly the no flux condition at $x = 1$ (Equation (5)) is implemented in cell N as:

$$\begin{aligned}\frac{\Delta x}{\Delta t}\left(\rho_N^{n+1}-\rho_N^n\right) + D_{N-\frac{1}{2}}\frac{\left(\rho_N^{n+1}-\rho_{N-1}^{n+1}\right)}{\Delta x}\\ - \frac{1}{2}\chi_{N-\frac{1}{2}}^n\frac{\left(\alpha_N^n-\alpha_{N-1}^n\right)}{\Delta x}\left(\rho_N^n-\rho_{N-1}^n\right) + \frac{1}{2}\chi_{N-\frac{1}{2}}^n\frac{\left|\alpha_N^n-\alpha_{N-1}^n\right|}{\Delta x}\left(\rho_N^n-\rho_{N-1}^n\right) = \Delta x\xi\rho_N^n. \qquad \text{(S4)}\end{aligned}$$

At each timestep we check the CFL stability condition for the upwinding scheme given by

$$\frac{\Delta t}{\Delta x}\chi_{i+\frac{1}{2}}|\alpha_{i+1}-\alpha_i| \leq 1. \tag{S5}$$

We use a simple adaptive scheme such that if the CFL condition is not met, the timestep of the solver is sufficiently reduced.

Having updated ρ, we then solve Equation (4) for the cytokine concentration α in cells $i = 2, ..., N-1$ using an implicit scheme for the diffusion term:

$$\begin{aligned}\frac{\Delta x}{\Delta t}\left(\alpha_i^{n+1}-\alpha_i^n\right) - D_{i+\frac{1}{2}}\frac{\left(\alpha_{i+1}^{n+1}-\alpha_i^{n+1}\right)}{\Delta x} + D_{i-\frac{1}{2}}\frac{\left(\alpha_i^{n+1}-\alpha_{i-1}^{n+1}\right)}{\Delta x}\\ = \Delta x\left(\eta_1 \mathbb{1}_{\{x\leq x_t\}} + \eta_2\rho_i^{n+1}\mathbb{1}_{\{x\geq x_t\}} - \kappa\rho_i^{n+1}\alpha_i^n - \nu\alpha_i^n\right).\end{aligned} \tag{S6}$$

The boundary condition at $x = 0$ (Equation (7)) is implemented in cell 1 as:

$$\begin{aligned}\frac{\Delta x}{\Delta t}\left(\alpha_1^{n+1}-\alpha_1^n\right) - D_{\frac{3}{2}}\frac{\left(\alpha_2^{n+1}-\alpha_1^{n+1}\right)}{\Delta x} + \Delta x D_1\zeta\alpha_1^{n+1}\\ = \Delta x\left(\eta_1 \mathbb{1}_{\{x\leq x_t\}} + \eta_2\rho_1^{n+1}\mathbb{1}_{\{x\geq x_t\}} - \kappa\rho_1^{n+1}\alpha_1^n - \nu\alpha_1^n\right).\end{aligned} \tag{S7}$$

The no flux condition at $x = 1$ (Equation (5)) is implemented in cell N as:

$$\begin{aligned}\frac{\Delta x}{\Delta t}\left(\alpha_N^{n+1}-\alpha_N^n\right) + D_{N-\frac{1}{2}}\frac{\left(\alpha_N^{n+1}-\alpha_{N-1}^{n+1}\right)}{\Delta x}\\ = \Delta x\left(\eta_1 \mathbb{1}_{\{x\leq x_t\}} + \eta_2\rho_N^{n+1}\mathbb{1}_{\{x\geq x_t\}} - \kappa\rho_N^{n+1}\alpha_N^n - \nu\alpha_N^n\right).\end{aligned} \tag{S8}$$

The initial condition for α is determined by solving the numerical scheme in Equations (S6)-(S8) until a steady state is reached.

2 Model parameters

A description of all model parameters and their corresponding units are given in Table S1. While, for convenience, the mathematical model is defined and solved on the domain $x \in [0, 1]$, we note that the solution may be rescaled to correspond to the dimensions of the Organoplate and recover dimensional parameters. The parameter values used for the numerical simulation shown in Figure 5 are listed in Table S2.

3 Optimisation algorithm details

The parameter optimisation framework used in this paper and summarised in Algorithm 1 (main text) leverages Kriging models as a surrogate for the solution of the full mathematical model. Kriging, also known as Gaussian process modelling, is a method of statistically interpolating data to build a response surface [3]. Central to Kriging is the assumption that the sampled points of the true function may be realised as a stochastic process with mean zero plus a trend function, often assumed to be simply a constant. The stochastic process is assumed to have a spatial correlation function such that the correlation between points is related to the distance between them. Due to this statistical formulation, Kriging models also provide an uncertainty of the Kriging estimate at each point making them a popular choice for surrogate-based optimisation algorithms [1, 3]. A more detailed, technical explanation of Kriging methods may be found in the aforementioned references.

There are numerous modifications to the original Kriging-based optimisation algorithm of Jones *et al.* [1] which have been proposed in order to improve its speed, convergence and parameter space exploration properties. Numerical schemes used for simulating the solution of a mathematical model typically place conditions upon the parameters of the numerical scheme which must

Parameter	Description	Units
$\bar{D}_\rho,\ \bar{D}_\alpha$	Diffusivity of species in ECM	$\mu m^2/h$
$\bar{\chi}$	Chemotactic sensitivity coefficient in ECM	$\mu m^2/h$
$\mu_\rho,\ \mu_\alpha$	Relative motility of species in tissue scaling factor	*dimensionless*
k	Chemokine receptor affinity	nM
ξ	Outflux rate of cells from region of interest	$1/h$
η_1	Rate of chemoattractant production by macrophages	nM/h
η_2	Rate of target engagement-induced chemoattractant production	$(nM/h)/(cell/\mu m)$
κ	Chemoattractant uptake rate	$nM/(cell/\mu m)$
ν	Chemoattractant degradation rate	$1/h$
$\tilde{\rho}$	Density of source of infiltrating T cells	$\#cells/\mu m$
ζ	Diffusive flux of chemoattractant at $x = 0$	$1/\mu m$

Table S1: Summary of all model parameters in Equations (1)-(9).

Parameter	Value
$\bar{D}_\rho$	8.79 x 10^{-4}
$\bar{D}_\alpha$	0.28
$\bar{\chi}$	0.51
μ_ρ	17.44
μ_α	10.84
k	0.8
ξ	0.42
η_1	0.55
η_2	0.44
κ	0.23
ν	0.01
$\tilde{\rho}$	74.23
ζ	0.08

Table S2: List of model parameter values used for the numerical simulation shown in Figure 4.

**Parameter**	Description	**Value**
$iter_{min}$	Minimum number of domain size reductions	4
tol	Convergence threshold as percentage of previous optimum	1%
p_{init}	Initial sample size	$10x$\{#model parameters\}
p_{max}	Total number of simulations per iteration	300
k	Number of parallel sample points	3
l	Number of local sampling points	3

Table S3: List of parameter values used for the optimisation Algorithm 1.

be satisfied to guarantee stability and convergence to the true solution. Such constraints may be often be overcome in an adaptive manner by reducing the time step or mesh size in the spatial discretization. However, doing so is likely to impact significantly the computational cost required for the simulation. For our purposes, we anticipate that the true solution should be well-behaved. That is, we would not expect a solution which blows-up or which becomes singular or zero everywhere. As such, we place a limit on the mesh refinements allowed for the stability of the numerical scheme, and deem regions of parameter space which do not satisfy the stability conditions as 'infeasible'. We note that such regions may not be determined *a priori*. To handle such regions of parameter space we use the approach of Sacher *et al.* [2] whereby a separate support vector machine model is built in addition to the Kriging model as a classifier to determine feasible regions of parameter space in which to sample (step 2.2 in Algorithm 1 of the main text).

Another common limitation of Kriging-based approaches is the lack of parallelisation due to the need for sampling new points one at a time after updating the surrogate model. This further hinders the performance of the algorithm since the computational cost of the constructing the Kriging model scales with the total number of points in the sample and, as such, the progress of the algorithm becomes slower as it progresses. We follow the approach of Zhan *et al.* [6] and maximise the pseudo-EI criterion in place of the true EI function in order to take advantage of parallelisation and solve the model at multiple points in parameter space simultaneously (step 2.3 in Algorithm 1 of the main text). Parallelisation in this manner may be viewed as a compromise, balancing a greater number of sampled points with a potential decrease in optimality of the proposed parameter sets to sample.

For high-dimensional parameter spaces, many samples may still be required to optimally explore the space. Sampled points may also be far away from each other leading to poor estimates of the Kriging correlation. As such we incorporate a local sampling around each new point as proposed by Xing *et al.* [4]. These processes are then iterated until the total budget for model simulations, p_{max}, is reached (step 2 in Algorithm 1 of the main text).

We also follow Xing *et al.* [4] and iteratively restrict the size of the parameter space to improve convergence of the algorithm to the optimum and avoid excessive exploration of the parameter space. Iterative reduction of the domain size proceeds until a pre-determined convergence threshold is met and a minimum number of domain size reductions have occurred (step 3 in Algorithm 1 of the main text). The parameter ranges for each parameter are scaled to $[0, 1]$ for optimisation such that each dimension is explored equally.

The optimisation algorithm is implemented in MATLAB and adapted from an existing open source implementation [5] of a parallel version of the orginal algorithm by Jones *et al.* [1]. A list of parameter values used for the optimisation algorithm is given in Table S3.

References

[1] D. R. Jones, M. Schonlau, and W. J. Welch. Efficient Global Optimization of Expensive Black-Box Functions. *Journal of Global Optimization*, 13:455–492, 1998. doi: https://doi.org/10.1023/A:1008306431147.

[2] M. Sacher, R. Duvigneau, O. Le Maître, M. Durand, É. Berrini, F. Hauville, and J. A. Astolfi. A classification approach to efficient global optimization in presence of non-computable domains. *Structural and Multidisciplinary Optimization*, 58(4):1537–1557, oct 2018. ISSN 16151488. doi: 10.1007/s00158-018-1981-8. URL `https://doi.org/10.1007/s00158-018-1981-8`.

[3] J. Sacks, W. J. Welch, T. J. Mitchell, and H. P. Wynn. Design and Analysis of Computer Experiments. *Statistical Science*, 4(4):409–423, nov 1989. ISSN 0883-4237. doi: 10.1214/ss/1177012413. URL `http://projecteuclid.org/euclid.ss/1177012413`.

[4] J. Xing, Y. Luo, and Z. Gao. A global optimization strategy based on the Kriging surrogate model and parallel computing. pages 405–417, 2020. doi: https://doi.org/10.1007/s00158-020-02495-6.

[5] D. Zhan. Github repository: Single objective EGO algorithms, 2020. URL `https://github.com/zhandawei/Single_objective_EGO_algorithms`.

[6] D. Zhan, J. Qian, and Y. Cheng. Pseudo expected improvement criterion for parallel EGO algorithm. *Journal of Global Optimization*, 68(3):641–662, jul 2017. ISSN 15732916. doi: 10.1007/s10898-016-0484-7. URL `https://link.springer.com/article/10.1007/s10898-016-0484-7`.
